# Impact of the Management and Proportion of Lost to Follow-Up Cases on Cancer Survival Estimates for Small Population-Based Cancer Registries

**DOI:** 10.1155/2022/9068214

**Published:** 2022-01-30

**Authors:** Fabian Gil, Adalberto Miranda-Filho, Claudia Uribe-Perez, N. E. Arias-Ortiz, M. C. Yépez-Chamorro, L. M. Bravo, Esther de Vries

**Affiliations:** ^1^PhD Program in Clinical Epidemiology, Department of Clinical Epidemiology and Biostatistics, Pontificia Universidad Javeriana, Bogotá, Colombia; ^2^Department of Clinical Epidemiology and Biostatistics, Faculty of Medicine, Pontificia Universidad Javeriana, Bogotá, Colombia; ^3^Cancer Surveillance Branch, International Agency for Research on Cancer, Lyon, France; ^4^Population Registry of Cancer of the Metropolitan Area of Bucaramanga, Genetic Study of Complex Diseases Research Group, Universidad Autónoma de Bucaramanga, Bucaramanga, Colombia; ^5^Population Registry of Cancer of Manizales, Health Promotion and Disease Prevention Research Group (Grupo de Investigación Promoción de la Salud y Prevención de la Enfermedad-GIPSPE) Universidad de Caldas, Manizales, Colombia; ^6^Cancer Registry of Pasto, Centro de Estudios en Salud (CESUN), Facultad de Ciencias de la Salud, Universidad de Nariño, Colombia; ^7^Cancer Registry of Pasto, Centro de Estudios en Salud (CESUN), Universidad de Nariño, Colombia

## Abstract

**Background:**

Estimation of survival requires follow-up of patients from diagnosis until death ensuring complete and good quality data. Many population-based cancer registries in low- and middle-income countries have difficulties linking registry data with regional or national vital statistics, increasing the chances of cases lost to follow-up. The impact of lost to follow-up cases on survival estimates from small population-based cancer registries (<500 cases) has been understudied, and bias could be larger than in larger registries.

**Methods:**

We simulated scenarios based on idealized real data from three population-based cancer registries to assess the impact of loss to follow-up on 1-5-year overall and net survival for stomach, colon, and thyroid cancers—cancer types with very different prognosis. Multiple scenarios with varying of lost to follow-up proportions (1-20%) and sample sizes of (100-500 cases) were carried out. We investigated the impact of excluding versus censoring lost to follow-up cases; punctual and bootstrap confidence intervals for the average bias are presented.

**Results:**

Censoring of lost to follow-up cases lead to overestimation of the overall survival, this effect was strongest for cancers with a poor prognosis and increased with follow-up time and higher proportion of lost to follow-up cases; these effects were slightly larger for net survival than overall survival. Excluding cases lost to follow-up did not generate a bias on survival estimates on average, but in individual cases, there were under- and overestimating survival. For gastric, colon, and thyroid cancer, relative bias on 5-year cancer survival with 1% of lost to follow-up varied between 6% and 125%, 2% and 40%, and 0.1% and 1.0%, respectively.

**Conclusion:**

Estimation of cancer survival from small population-based registries must be interpreted with caution: even small proportions of censoring, or excluding lost to follow-up cases can inflate survival, making it hard to interpret comparison across regions or countries.

## 1. Introduction

Population-based cancer registries (PBCR) are the gold standard source of population-based incidence and survival statistics [1, 2]. Estimation of survival requires follow-up of a cohort of cancer patients until their moment of death or study closure, ensuring complete follow-up and good quality data. Many PBCR, contrary to the situation of clinical trials, do not perform routine follow-up of all patients when they perform survival analyses. Usually, they cross-link information from civil registration and vital statistics of their population to be informed regarding date (and cause) of death. If the completeness or quality of the vital statistics is suboptimal, deceased patients may not be reported and therefore considered erroneously being alive at the date of administrative follow-up. The quality of the death-registry is relatively complete in Colombia [3]; therefore, absence of a death certificate is usually considered as an indicator to consider the patient alive. However, in Colombia and low- and middle-income countries (LMIC), this cross-linking between registries and vital statistics is difficult and often only possible for the regional vital statistics data—if a patient died in another city, the registries would not be notified. Sometimes strict personal data protection policies [4] inhibit this linkage altogether, and registries have to rely on notes made by registrars when coincidentally encountering patients already in the registry.

In cohort studies of time-to-event data, lost-to-follow-up (LFU) is a critical source of bias [5] that threaten internal validity of estimates and can compromise survival estimates. Differences in cancer registry processes and follow-up practices have been assessed as being responsible for observed cancer survival differences [6–8]. Previous studies have reported the impact of incomplete registration and inclusion of death-certificated-only (DCO), death-certificate-notified (DCN), and death certificate-initiated (DCI) cases [6, 9–11] and others on the impact of completeness of follow-up over cancer survival [7, 8, 12–15] employing real data from PBCR and hospital populations or simulations. However, the studies that evaluated impact of LFU on survival considered sample sizes over ≥1,000 cases, whereas PBCR in LMIC are usually regional with relatively small sizes (populations of around 300,000 to 1.5 million inhabitants, implying less than 500 cancer cases for individual cancer during a 5-year period) [16], and there is no information regarding the impact in such situations with few cases. Studies with simulated data artificially create a “perfect registry” where the artificial truth is known; however, these do not necessarily mimic demographic patterns, and their relations with prognoses that are similar to “real-life” situations and LFU patterns could be hard, and conclusions could be nonspecific. The previous studies based on real data applied a set of scenarios of percentages of LFU on real registry data and evaluated a point estimation of effect over survival estimations.

For this work in particular, we defined LFU patients to be those patients for whom vital status was not available (neither actively reported as alive nor deceased) through administrative follow-up, and there is no date of last contact through registrar notes. Dealing with such LFU cases is hard, there is no clear censor date, and one may either argue to delete them from analyses because of incomplete follow-up or to consider them alive at the end of the study. We hypothesized that even when such LFU events are rare, in small PBCR, they may have a stronger impact on net survival estimation than in larger PBCR; because of the very small number of observations, particularly within subgroups, any loss may substantially influence survival estimations.

In this study, we used real-world data from small PBCR to evaluate, through simulations, the potential bias that censoring or excluding LFU cases have on 1-, 3-, and 5-year overall and net survival. The simulated scenarios include cancers of good, medium, and poor prognosis, and different percentages of LFU, with distinct sample sizes but all under 500 cases. Bootstrap confidence intervals are presented for the mean of the bias in the evaluated scenarios to facilitate the interpretation of the estimates obtained.

## 2. Materials and Methods

To evaluate the size of the potential bias that censoring versus excluding cases LFU has on estimates of all-cause (overall) and net survival in PBCR, we performed multiple simulations of distinct scenarios. The simulations employed real data from three Colombian good quality PBCR: Bucaramanga metropolitan area, Manizales, and Pasto; age-standardized incidence rates for all cancers, all ages, and both sexes for this PBCR ranged from 162.3 to 181.8 per 100,000 person year in 2012 [17]. We created a dataset combining data from these PBCR including all incident first primary cases diagnosed between 2008 and 2012 of cancers of the stomach (ICD-O-3 topography: C16.0–C16.9), colon (C18.0-C18.9), and thyroid gland (C73.9) occurring in persons aged between 18 and 99 years. We excluded cases notified by death certificate only (DCO), cases with missing date of cancer diagnosis and cases without follow-up. Cases with a follow-up time of zero (occurring when diagnostic details based on pathology specimens taken during life were delivered after the date of death) were replaced with a follow-up time of 1 day to differ these cases from DCO cases (where the diagnosis is only based on the death certificate and therefore follow-up time is by definition 0 [6]. This database represents a near “perfect” scenario because follow-up is complete for all included cases by 5 years from diagnosis, and their vital status is known, with demographic and survival characteristics more representative of South America. However, the data are not completely representative, because some cases were excluded (table S1).

The strategy to select the three types of cancer was based on their prognosis and relevance for our population [18]: poor (stomach), medium (colon), and good (thyroid gland) prognosis.

For each cancer type, we simulated 45 (3 × 5 × 3) scenarios varying in sample size, % of cases randomly created to be LFU, and survival time used for the estimation. Sample sizes (SS) of 100, 300, and 500 were used to represent the PBCR's size based on 5 years observed incident number of cases. We modelled the percentage of cases LFU (PLT) to be 1%, 5%, 10%, 15%, and 20%, and estimates were produced for survival time (ST) at one, three, and 5 years from the date of diagnosis. Each scenario was simulated 10,000 times.

**Figure 1:**
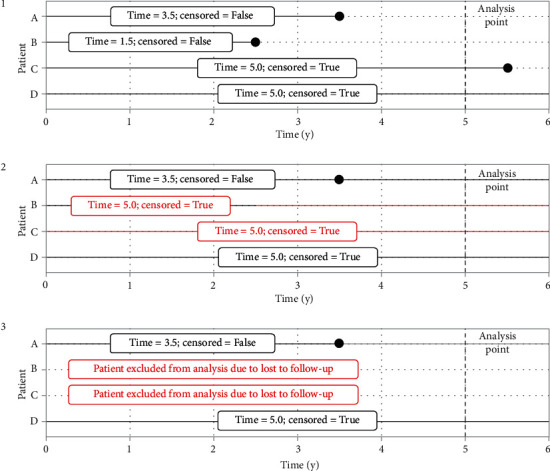
Schematic representation of the steps of the simulation process regarding simulating loss to follow-up. Time until death for four hypothetical cancer patients in this study; horizontal lines represent time from diagnosis with dots representing the date of death; the labels projected on top of the lines convey information regarding time under observation or to event in a 5-year survival analysis depending on the exact simulation step. (1) Complete cases, no simulation applied; (2) censured case scenario: randomly patients B and C were censored to be lost (simulating date of death was not reported by vital statistics, and therefore, patient erroneously considered alive at analysis point); time of patient B changed from 2.5 to 5.0 years, time of patient C unchanged by simulation as patient really did not decease; (3) excluded cases scenario: patients B and C in previous step changed to be lost to follow-up were excluded from analysis.

Three simulation steps were performed for each scenario (see Figure 1):

*Complete cases (reference scenario)*: a random sample of size ss with replacement was taken from the cases with complete and known follow-up (deaths occurring at any time and alive with follow-up time greater or equal to ST).

*Censored cases*: from the sample created in step 1, a random sample of PLT without replacement was modelled to be LFU, and these patients were censored at time ST. Loss to follow-up was simulated independently of real vital status and time from diagnosis.

*Excluded cases*: the cases modelled to be LFU in the previous step were deleted from the dataset and excluded from the survival analysis.

The Kaplan-Meier survival [19] estimation and the net survival estimation using the Pohar-Perme method [20] were calculated on the datasets created in each simulation step. For net survival, complete life tables by sex and for single years (period 2008-2018) for each PBCR were used; the life tables were obtained from the Colombian National Administrative Department of Statistics–DANE [21]. No age-standardization was performed.

The performance measure was bias on the Kaplan-Meier and net survival estimates due to censoring or excluding LFU cases. This was calculated as the difference between the estimators based on complete cases and censored cases or complete cases and excluded cases. The relative bias was calculated as bias divided by the estimator based on complete cases (reference scenario). A data set with 10,000 relative differences was obtained for each of the 45 (3 distinct SS, 5 distinct PLF, and 3 distinct ST) simulated scenarios.

For each scenario, we obtained central tendency and variability statistics for the relative bias, as well as 95% empirical bootstrap confidence intervals for the relative bias [22].

Data management was performed using R version 4.0.3 [23], and data analysis was performed using Stata version 16.2 [24] employing the command strs [25].

## 3. Results

Records of 3,455 incident cancer patients were collected from the three PBCR, 1,528 (44.2%) for stomach, 902 (26.1%) for colon, and 1,025 (29.7%) for thyroid gland cancer. After applying the exclusion criteria, 2,924 cases remained: 1,188 for stomach, 759 for colon, and 977 for thyroid gland cancer (details for reasons for exclusion in table S1). The proportion of excluded tumors was 22.3% for stomach, 15.9% for colon, and 4.7% for thyroid gland. The mean age for stomach cancer cases was 63.8 years (standard deviation (SD): 15.0), for colon 64.8 years (SD 14.1), and for thyroid gland 48.4 years (SD 14.3). The proportion of women was 41.5, 60.5, and 86.4% for stomach, colon, and thyroid gland, respectively.

Table 1 shows overall one-, three-, and 5-year all-cause and net survival for the complete case analysis of the three cancer types (reference scenario). For stomach cancer, one-year overall and net survival were 0.39 and 0.40; corresponding figures were 0.66 and 0.67 for colorectal cancer and 0.96 and 0.96 for thyroid cancer. Five-year overall and net survival were 0.19 and 0.21 for stomach cancer, 0.44 and 0.50 for colon cancer, and 0.92 and 0.95 for thyroid gland cancers, respectively. Net survival was always slightly higher than overall survival.

### 3.1. Censoring Lost to Follow-Up

#### 3.1.1. Overall Survival

**Figure 2:**
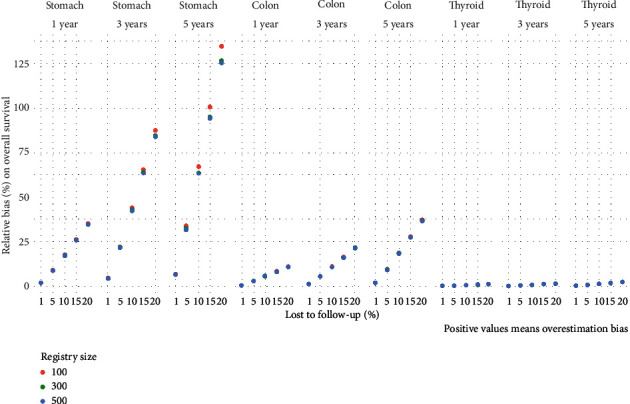
Relative bias (%) on overall survival due to censoring lost to follow-up cases by cancer localization, follow-up time, percentage lost to follow-up, and registry size.

The simulation exercise showed that censoring LFU patients always leads to an overestimation of the one-, three-, and 5-year overall survival for the three cancers (Figure 2, Table S2).

The degree of overestimation depended on the overall prognosis of the type of cancer and simulated time-to-event: overestimation of survival for thyroid gland cancer (very good prognosis) had a small effect (range 0.0–2.3 percentage points); the maximum value was observed at 5-year survival with a 20% LFU. For colon cancer (intermediate prognosis), the effect was moderate for one-year survival estimates when the percentage of LFU was up to 10% (range 0.0-5.3 percent points); the same was observed in three-year survival with LFU proportions of 5% and in 5-year survival with LFU of 1%. Stomach cancer survival (poor prognosis) was most affected with 1.7–34.9, 4.2–87.4, and 6.3–134.5 percentage points overestimated survival at one-, three-, and 5-year overall survival upon censoring LFU patients. The bias was moderate for colon cancer survival estimates (medium prognosis) with 0.5–9.6 percent point overestimation of survival along one- to 5-year survival. The lowest bias was observed for thyroid gland cancer (good prognosis) with 0.0–0.9 percent point overestimation of survival in all cases simulated.

The empirical distribution of the relative bias in overall survival due to censoring LFU cases was symmetric. 95% confidence intervals (table S3) show the effect of sample size and LFU proportion, low sample size, and high LFU proportion logically resulting in the lowest precision.

#### 3.1.2. Net Survival

**Figure 3:**
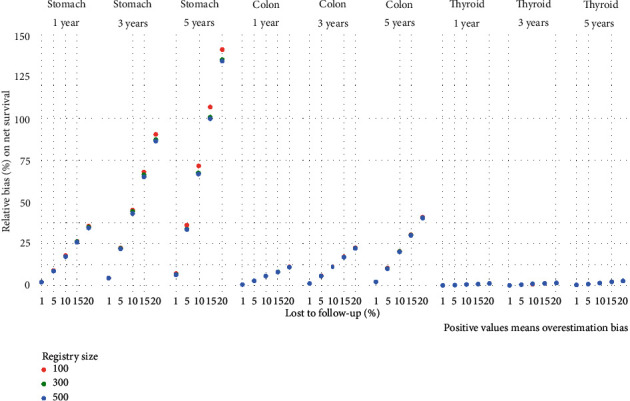
Relative bias (%) on net survival due to censoring lost to follow-up cases by cancer localization, follow-up time, percentage lost to follow-up, and registry size.

As in overall survival, net survival was overestimated in all simulated scenarios where censored cases were included in analyses (Figure 3, Table S4); on average, the relative bias of censoring patients LFU was small for thyroid gland cancer even with high proportions of LFU and longest follow-up (0.1–2.6 percentage points at 5-year survival). For colon cancer, the relative bias was also small at one- and three-year survival with LFU ratio of 10% and 5-year survival of 5% cases simulated to be LFU (range 0.0–5.6 percentage points). However, with 5% or more cases being simulated to be LFU in estimating 5-year survival, the impact was substantial (range 10.0–41.0 percent points). Censoring had the biggest relative bias on stomach cancer survival estimates: even when randomly censoring 5%, one- and three-year survival was affected between 8.6 and 90.4 percent points; influence on 5-year survival ranged between 6.7 (for 1% censored) and 143.3 percentage points.

95% confidence intervals for the average relative bias due to censoring LFU are shown in table S5, the empirical distribution was symmetric around the mean, and the variability increases with small registry size large LFU ratio and time to follow-up; these same factors affected precision of confidence intervals.

### 3.2. Excluding LFU

**Figure 4:**
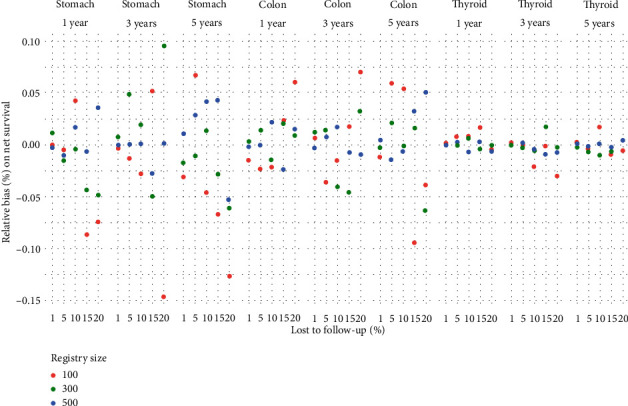
Relative bias (%) on overall survival due to excluding lost to follow-up cases by cancer localization, follow-up time, percentage lost to follow-up, and registry size.

**Figure 5:**
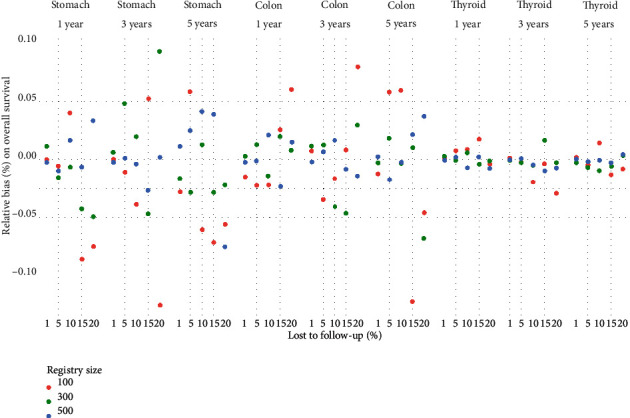
Relative bias (%) on net survival due to excluding lost to follow-up cases by cancer localization, follow-up time, percentage lost to follow-up, and registry size.

In our study, elimination of LFU cases did not impact overall nor net survival independently of follow-up time, percent of lost to follow, and cancer localization (Figures 4 and 5, tables S2 and S3). The empirical distribution of the relative bias was symmetric around zero; the dispersion was inverse to the size of the registry and proportional to time to follow-up and proportion LFU. Tables S4 and S5 show 95% confidence intervals for the relative bias on overall and net survival, respectively. Unlike censoring, which exclusively leads to overestimation of estimates, excluding LFU can lead to under- or overestimation of net and overall survival estimations.

## 4. Discussion

Our results show that censoring the LFU cases leads to an overestimation of survival. The absolute and relative magnitude of this bias is greater in cancers with a poor prognosis and with a longer follow-up time, and this effect is slightly greater for net survival compared to overall survival. On the other hand, excluding LFU had, on average, null effect on both overall and net survival, but the confidence intervals show that survival can be underestimated or overestimated; this effect was greater in cancers with a worse prognosis, high percentage of LFU, and small registry size.

Colombia, like most LMIC, does not have national PBCR, the regional PBCR cover together less than 15% of the population [16], and their source populations are relatively small. Strict personal data protection policies pose difficulties in linking vital statistics data with those from the PBCR [4], a situation shared with many LMIC. When routine data linkage is not possible, RCBP must perform active follow-up or do very strong lobbying with regional institutions to obtain the vital status and date of death of patients, facing problems such as the mobility of patients (if they move to a different city, regional vital statistics will not show the vital status nor date of death), change or lack of medical insurance, and changes of attending hospitals, which produces LFU in active follow-up. Previous studies have shown low to moderate bias on survival estimates on big national PBCR or simulations with sample sizes over 1000 cases where LFU cases could have a modest impact over estimations [7, 8, 12], but we supposed that in small PBCR, the impact could be a problem on survival estimates because of the very small number of observations and the potentially larger impact of losing some of them.

Dealing with LFU on survival analysis requires making decisions on the statistical analysis: censoring or excluding LFU cases. Censoring LFU would assume that the censured subject was alive at the moment of censoring and had the same survival probabilities as the other subjects still alive at that moment. As censoring is likely to be either due to moving out of the area (probably implying good health or precisely the opposite: people may migrate towards larger cities to have medical attention because of a worse health) or having passed away (very poor prognosis), this assumption is obviously not realistic and logically produces an overestimation of overall and net survival if the LFU subject had previously died, but the death was not reported, or if the subject was at increased risk of dying shortly after censoring. The problem is that frequently, one does not know a patient is LFU—if the linkage with vital statistics or the quality of vital statistics is suboptimal, patients may seem “alive” in the linkage because their death was not reported or was reported in a different municipality and as mentioned before, some registries only have access to vital statistics from their own municipality. Complete follow-up of registered cancer patients is an important aspect of data quality; therefore, PBCR should be encouraged to advocate for access to national data, help improve vital statistics quality, and try to perform routine trace-back death-certificate-only cases assessing for previous diagnosis [10]—not doing this results in unnecessary high proportions of DCO and inflated survival. Also, although PBCR in the region suffer from very limited personal and financial resources in PBCR [26], they should keep registries of last known date of follow-up for patients for whom no date of death is known. This information must be used in survival analysis as a better approach to deal with LFU; in other cases, patients could be assumed alive forever. Additionally, registries may consider to formulate “rules” of assuming patients who cannot be linked as having died after a certain age (e.g., 110 years) or after a certain number of years after diagnosis in situations where linkage with or quality of vital statistics is known to be incomplete. In particular for cancers with poor prognosis, the latter option may be reasonable, but these should be decisions made on the individual situation of each cancer registry.

In our study, censoring LFU cases in small sample sizes overestimated overall survival in a similar direction as previous studies with large sample sizes [12, 14, 15] but with a higher magnitude and variability of overestimations. The observed impact on net survival also was larger than reported from studies with sample size > 1,000 cases, even for cancers with a medium to good prognosis cancers [12, 13]. Probably, the smaller base for estimations causes this larger impact.

To our knowledge, this is the first study to explore the effect of completeness of follow-up of cases in small PBCR to evaluate deviations in survival estimations. A previous study evaluated bias on overall survival due to LFU cases in hospital-based cancer registries in Japan with sizes over 500 cases; our study differs in evaluating both overall and net survival in population-based settings and smaller numbers of cases (100-500) and moreover in a Middle Income Country, with probably different realities in terms of quality and completeness of vital statistics registries [14].

The main strength of our study is to have quantified the effect of excluding LFU cases, an approach never recommended in practice but sometimes applied at the moment of data analysis. Our simulation involved 10,000 datasets using real data allowing us to evaluate the random error of the bias, and bootstrap CIs are reported for the average of the bias produced in the estimation of overall and net survival in each of the studied scenarios. Using real South American databases is an advantage—even though these kinds of data issues exist throughout the globe, they may be of different magnitudes in LMIC. PBCR in LMIC are maturing and increasingly realizing survival analyses—these results are important for local evaluations. We decided not to estimate the bias on survival estimation for all cancers combined, because cancer is the first leading cause of premature death before the age of 70 in Colombia [27], and using all cancers combined would violate an important assumption of net survival methods: the number of cancer deaths must be a negligible amount of the all-cause deaths in the general population [28].

A possible weakness of our study includes our LFU to be modelled to occur completely at random, but we argue that this scenario is reasonable in our context because in adult cancer patients linking problems of official vital statistics with PCBR registries does not depend on patient characteristics (it is unlikely that vital statistics registration is more reliable for certain sex, age, or cancer stage groups). LFU in other situations has been reported to be more frequent in older subjects or differ by sex [8, 14, 15] but inspection of our PBCR data did not show distinct patterns of LFU by age were observed. We noted some differences in LFU by sex, but these were different and opposed in direction depending on cancer type. We simulated scenarios with different percentages of LFU by sex, and the results were very similar to those scenarios with random LFU (results not shown). Other studies are required to evaluate the effect of differential LFU by other variables. It is likely that, depending on the exact scenario, in this case, the effect of censoring LFU could be greater than those observed in our study.

We called the differences between complete cases and censoring/deleting case estimates “bias”—but we do not know the true survival proportion at each timepoint. Our estimations came from two samples (one under a real case, and one under a modified case), but we cannot compare back to the true survival—both are estimated with error. Therefore, in the strict sense of the word, this difference is an error or deviation rather than bias, but the term bias is more commonly understood.

The proportion of cases excluded for this exercise because of follow-up (table S1) was higher for cancers with a poor prognosis. This reflects the real situation of patients for who sometimes the diagnosis comes after their moment of death or who are diagnosed postmortem. Moreover, such patients may quickly be referred to highly specialized hospitals in other regions and lost to active follow-up. It is important to remember that the database constructed for this work does not represent a real population to which the survival results can be extended because of the exclusions, but as the objective of this work was to quantify the effect of LFU, we feel this should not represent a problem.

Thyroid cancer was included because of its very high incidence and good prognosis, which differs between sexes [29]. Overdiagnosis exists in Colombia but probably to a lower extent than in certain high-income countries [30]. Breast cancer was not considered: screening for this tumor is offered in the Colombian health system, but its uptake and the quality of the mammograms and their lectures are quite social-class dependent, probably causing length biases differentially between social groups [31]. As the aim of this study was to grasp the impact and the magnitude of the impact of LFU on survival estimates, rather than to estimate specific cancer survival, we feel that thyroid cancer provides an interesting scenario.

Difficulties in linking date of death and vital status to PBCR incidence data lead to LFU and thereby affect the validity and precision of estimates of overall survival and net survival, particularly when local policies do not allow or hinder linked vital statistics data with PBCR records. Random censoring LFU cases in the analyses leads to an overestimation of survival even with low percentages of LFU and is not recommended for cancers with a poor prognosis and even less for long periods of follow-up time. For cancers of medium and good prognosis, overestimations, on average, could be of small magnitude and even have a negligible effect. On the other hand, deleting LFU in the analysis on average showed no effect on the survival estimates (average bias zero), although the point estimates may be either underestimated or overestimated. These observed effects were stronger for net survival than overall survival and increased with increasing percentage of LFU and decreasing size of the PBCR.

It is important to interpret survival estimates from small PBCR even with small LFU proportions with caution, particularly when comparing survival between regions or countries. Percentages of LFU (if known) should always be reported with the way LFU cases were handled in analysis, particularly when studies combine data from different PBCRs, variations in LFU the impact of bias and random variations that these have on the point estimates. Ideally, the authors would also include a description of the quality of vital statistics in their region. PBCR should be allowed to record-linkage with vital statistics to improve data quality and survival estimates.

## 5. Conclusions

Randomly censoring cases to be lost to follow-up leads an overestimation of overall and net survival; the effect of this censoring increases with survival time studied and proportion of cases censored and was more pronounced for types of cancer with poor prognosis. Registry size influences the dispersion of the impact: small registry size increases dispersion of impact over overall survival. It is important to grasp the size of this impact when interpreting estimates, and this study can help guide such exercises. Excluding LFU cases did not impact overall nor net survival on average, but random variations result in individual estimates which can either overestimate or underestimate both overall and net survival, and the confidence intervals of the survival estimates widen. Excluding cases should be never recommended in practice.

## Figures and Tables

**Table 1:** Numbers of complete cases available for analysis, number of cumulative deaths by time to follow-up, and 1-, 3-, and 5-year overall and net survival by cancer localization based on complete case analysis.

Cancer localization	*n*	Cumulative deaths (y)			Overall survival (y)			Net survival (y)		
		1	3	5	1	3	5	1	3	5
Stomach	1188	707	887	920	0.39	0.22	0.19	0.40	0.24	0.21
Colon	759	255	364	404	0.66	0.50	0.44	0.67	0.54	0.50
Thyroid gland	977	43	60	72	0.96	0.94	0.92	0.96	0.96	0.95

## Data Availability

The cancer patient data employed in this study are property of each PBCR, and restrictions apply to the availability of these data, which were used under license for the current study, and so are not publicly available. Individual registries (coauthors in this study) can evaluate reasonable requests for data on a one-by-one basis.

## Supplementary Materials

Supplementary Materials Supplementary Table S1 shows available cases, exclusion criteria, and remaining cases by cancer localization. Supplementary Tables S2 and S4 summarise relative bias on overall and net survival due to censoring and excluding LFU cases for the simulated scenarios. Supplementary Tables S3 and S5 show 95% empirical bootstrap confidence intervals for relative bias on overall and net survival for the simulated scenarios.
