# Supplementary material for: Impact of the Management and Proportion of Lost to Follow-Up Cases on Cancer Survival Estimates for Small Population-Based Cancer Registries
Source: J Cancer Epidemiol. 2022 Jan 30;2022:9068214. doi: 10.1155/2022/9068214 (PMC8818438; doi:10.1155/2022/9068214)
Supplement: Supplementary Materials — Supplementary Table S1 shows available cases, exclusion criteria, and remaining cases by cancer localization. Supplementary Tables S2 and S4 summarise relative bias on overall and net survival due to censoring and excluding LFU cases for the simulated scenarios. Supplementary Tables S3 and S5 show 95% empirical bootstrap confidence intervals for relative bias on overall and net survival for the simulated scenarios. [file 9068214.f1.docx]

**Contents**

**Table S1.** Cases available, exclusion criteria and cases remaining for analysis by cancer localization

**Table S2.** Summary of relative bias on 1-, 3- and 5- year overall survival due to censuring or excluding lost to follow-up by cancer localization, lost to follow-up percent, and registry size

**Table S3.** Empirical bootstrap confidence intervals (95%) for relative bias on overall survival due to censuring or excluding lost to follow-up by cancer localization, lost to follow-up percent, registry size, and follow-up time

**Table S4.** Summary of relative bias on 1-, 3- and 5- year net survival due to censuring or excluding lost to follow-up by cancer localization, lost to follow-up percent and registry size

**Table S5.** Empirical bootstrap confidence intervals (95%) for relative bias on net survival due to censuring or excluding lost to follow-up by cancer localization, lost to follow-up percent, registry size, and follow-up time

Table S1. Cases available, exclusion criteria and cases remaining for analysis by cancer localization

|  | | | | **Cancer localization** | |  | |
| --- | --- | --- | --- | --- | --- | --- | --- |
|  |  | Stomach |  | Colon | | Thyroid gland | |
|  | n |  | % | n % | | n % | |
| **Registered cases** | 1528 |  | 100 | 902 100 | | 1025 100 | |
| **Exclusion criteria** | | | | | | | |
| **Death certificate only** | 103 | 6.7% | | 45 | 5.0% | 12 | 1.2% |
| **Without date of diagnosis** | 3 | 0.2% | | 2 | 0.2% | 6 | 0.6% |
| **Without follow-up** | 234 | 15.3% | | 96 | 10.6% | 30 | 2.9% |
| **Remaining for analysis** | 1188 | 77.7% | | 759 | 84.1% | 977 | 95.3% |

**Table S2.** **Summary of relative bias on 1-, 3- and 5- year overall survival due to censuring or excluding lost to follow-up by cancer localization, lost to follow-up percent, and registry size**

Positive values mean overestimation bias

**1 year**

**Cancer localization**

**Censoring lost to follow-up Excluding lost to follow-up**

Lost to follow-up Lost to follow-up

1% 5% 10% 15% 20% 1% 5% 10% 15% 20%

Registry size mean SD mean SD mean SD mean SD mean SD mean SD mean SD mean SD mean SD mean SD

| **Stomach** | 100 | 1.7 | 1.4 | 8.7 | 3.5 | 17.5 | 5.5 | 26.1 | 7.5 | 34.9 | 9.1 | 0.0 | 1.3 | 0.0 | 3.1 | 0.0 | 4.4 | -0.1 | 5.6 | -0.1 | 6.7 |
| --- | --- | --- | --- | --- | --- | --- | --- | --- | --- | --- | --- | --- | --- | --- | --- | --- | --- | --- | --- | --- | --- |
|  | 300 | 1.7 | 0.8 | 8.5 | 2.0 | 17.1 | 3.1 | 25.6 | 4.1 | 34.2 | 5.2 | 0.0 | 0.8 | 0.0 | 1.7 | 0.0 | 2.6 | 0.0 | 3.2 | 0.0 | 3.8 |
|  | 500 | 1.7 | 0.6 | 8.5 | 1.5 | 17.0 | 2.4 | 25.6 | 3.2 | 34.1 | 3.9 | 0.0 | 0.6 | 0.0 | 1.3 | 0.0 | 1.9 | 0.0 | 2.4 | 0.0 | 2.9 |
| **Colon** | 100 | 0.5 | 0.7 | 2.7 | 1.7 | 5.4 | 2.5 | 8.1 | 3.2 | 10.9 | 3.7 | 0.0 | 0.7 | 0.0 | 1.7 | 0.0 | 2.4 | 0.0 | 3.1 | 0.1 | 3.7 |
|  | 300 | 0.5 | 0.4 | 2.7 | 1.0 | 5.3 | 1.4 | 8.1 | 1.8 | 10.7 | 2.1 | 0.0 | 0.4 | 0.0 | 1.0 | 0.0 | 1.4 | 0.0 | 1.8 | 0.0 | 2.1 |
|  | 500 | 0.5 | 0.3 | 2.7 | 0.8 | 5.4 | 1.1 | 8.0 | 1.4 | 10.7 | 1.7 | 0.0 | 0.3 | 0.0 | 0.8 | 0.0 | 1.1 | 0.0 | 1.4 | 0.0 | 1.6 |
| **Thyroid gland** | 100 | 0.0 | 0.2 | 0.2 | 0.5 | 0.5 | 0.7 | 0.7 | 0.9 | 0.9 | 1.0 | 0.0 | 0.2 | 0.0 | 0.5 | 0.0 | 0.7 | 0.0 | 0.9 | 0.0 | 1.1 |
|  | 300 | 0.0 | 0.1 | 0.2 | 0.3 | 0.5 | 0.4 | 0.7 | 0.5 | 0.9 | 0.6 | 0.0 | 0.1 | 0.0 | 0.3 | 0.0 | 0.4 | 0.0 | 0.5 | 0.0 | 0.6 |
|  | 500 | 0.0 | 0.1 | 0.2 | 0.2 | 0.5 | 0.3 | 0.7 | 0.4 | 0.9 | 0.4 | 0.0 | 0.1 | 0.0 | 0.2 | 0.0 | 0.3 | 0.0 | 0.4 | 0.0 | 0.5 |

**3 year**

**Censoring lost to follow-up**

**Excluding lost to follow-up**

| **Cancer localization** |  |  | Lost to follow-up |  |  |  |  | Lost to follow-up |  | |
| --- | --- | --- | --- | --- | --- | --- | --- | --- | --- | --- |
|  | 1% | 5% | 10% | 15% | 20% | 1% | 5% | 10% | 15% | 20% |

Registry size mean SD mean SD mean SD mean SD mean SD mean SD mean SD mean SD mean SD mean SD

| **Stomach** | 100 | 4.4 | 2.4 | 21.9 | 7.5 | 43.6 | 13.6 | 65.6 | 20.1 | 87.4 | 25.4 | 0.0 | 2.1 | 0.0 | 4.8 | 0.0 | 7.0 | 0.1 | 9.0 | -0.1 | 10.5 |
| --- | --- | --- | --- | --- | --- | --- | --- | --- | --- | --- | --- | --- | --- | --- | --- | --- | --- | --- | --- | --- | --- |
|  | 300 | 4.2 | 1.3 | 21.1 | 4.1 | 42.2 | 7.4 | 63.3 | 10.4 | 84.4 | 13.5 | 0.0 | 1.2 | 0.0 | 2.7 | 0.0 | 4.0 | 0.0 | 4.9 | 0.1 | 5.9 |
|  | 500 | 4.2 | 1.0 | 20.9 | 3.1 | 41.8 | 5.5 | 62.9 | 8.0 | 83.9 | 10.3 | 0.0 | 0.9 | 0.0 | 2.1 | 0.0 | 3.0 | 0.0 | 3.9 | 0.0 | 4.6 |
| **Colon** | 100 | 1.1 | 1.1 | 5.3 | 2.5 | 10.7 | 3.8 | 16.1 | 5.0 | 21.5 | 6.1 | 0.0 | 1.0 | 0.0 | 2.4 | 0.0 | 3.5 | 0.0 | 4.4 | 0.1 | 5.2 |
|  | 300 | 1.1 | 0.6 | 5.3 | 1.4 | 10.5 | 2.2 | 15.8 | 2.8 | 21.2 | 3.4 | 0.0 | 0.6 | 0.0 | 1.4 | 0.0 | 2.0 | 0.0 | 2.5 | 0.0 | 3.0 |
|  | 500 | 1.1 | 0.5 | 5.3 | 1.1 | 10.5 | 1.7 | 15.8 | 2.2 | 21.1 | 2.6 | 0.0 | 0.5 | 0.0 | 1.0 | 0.0 | 1.5 | 0.0 | 1.9 | 0.0 | 2.3 |
| **Thyroid gland** | 100 | 0.1 | 0.3 | 0.3 | 0.6 | 0.7 | 0.8 | 1.1 | 1.0 | 1.4 | 1.2 | 0.0 | 0.3 | 0.0 | 0.6 | 0.0 | 0.9 | 0.0 | 1.1 | 0.0 | 1.3 |
|  | 300 | 0.1 | 0.2 | 0.3 | 0.3 | 0.7 | 0.5 | 1.1 | 0.6 | 1.4 | 0.7 | 0.0 | 0.2 | 0.0 | 0.3 | 0.0 | 0.5 | 0.0 | 0.6 | 0.0 | 0.8 |
|  | 500 | 0.1 | 0.1 | 0.4 | 0.3 | 0.7 | 0.4 | 1.0 | 0.5 | 1.4 | 0.5 | 0.0 | 0.1 | 0.0 | 0.3 | 0.0 | 0.4 | 0.0 | 0.5 | 0.0 | 0.6 |

**5 year Censoring lost to follow-up Excluding lost to follow-up**

| **Cancer localization** | | |  | | |  | | Lost to follow-up | | | |  | | |  | | |  | | |  | | Lost to follow-up | | | |  | | | | | |
| --- | --- | --- | --- | --- | --- | --- | --- | --- | --- | --- | --- | --- | --- | --- | --- | --- | --- | --- | --- | --- | --- | --- | --- | --- | --- | --- | --- | --- | --- | --- | --- | --- |
|  | | | 1% | | | 5% | | 10% | | | | 15% | | | 20% | | | 1% | | | 5% | | 10% | | | | 15% | | | 20% | | |
|  | | Registry size | mean | | SD | mean | | SD | | mean | SD | mean | | SD | mean | | SD | mean | | SD | mean | | SD | | mean | SD | mean | | SD | mean | | SD |
| **Stomach** | | 100 | 6.7 | | 3.5 | 33.6 | | 12.9 | | 67.2 | 25.1 | 100.5 | | 36.1 | 134.5 | | 48.5 | 0.0 | | 2.6 | 0.1 | | 6.0 | | -0.1 | 8.6 | -0.1 | | 10.9 | -0.1 | | 13.0 |
|  | | 300 | 6.3 | | 1.8 | 31.6 | | 6.2 | | 63.3 | 11.9 | 94.8 | | 17.1 | 126.6 | | 22.3 | 0.0 | | 1.4 | 0.0 | | 3.4 | | 0.0 | 4.9 | 0.0 | | 6.0 | 0.0 | | 7.3 |
|  | | 500 | 6.3 | | 1.4 | 31.3 | | 4.9 | | 62.7 | 8.9 | 94.1 | | 13.2 | 125.2 | | 17.3 | 0.0 | | 1.1 | 0.0 | | 2.6 | | 0.0 | 3.7 | 0.0 | | 4.7 | -0.1 | | 5.6 |
| **Colon** | | 100 | 1.8 | | 1.4 | 9.2 | | 3.6 | | 18.5 | 5.7 | 27.5 | | 7.8 | 36.9 | | 9.6 | 0.0 | | 1.4 | 0.1 | | 3.2 | | 0.1 | 4.5 | -0.1 | | 5.8 | 0.0 | | 6.8 |
|  | | 300 | 1.8 | | 0.8 | 9.0 | | 2.0 | | 18.1 | 3.2 | 27.0 | | 4.3 | 36.1 | | 5.3 | 0.0 | | 0.8 | 0.0 | | 1.8 | | 0.0 | 2.6 | 0.0 | | 3.3 | -0.1 | | 3.9 |
|  | | 500 | 1.8 | | 0.6 | 9.0 | | 1.6 | | 18.0 | 2.5 | 27.0 | | 3.3 | 36.0 | | 4.1 | 0.0 | | 0.6 | 0.0 | | 1.4 | | 0.0 | 2.0 | 0.0 | | 2.5 | 0.0 | | 3.0 |
| **Thyroid gland** | | 100 | 0.1 | | 0.3 | 0.6 | | 0.8 | | 1.1 | 1.1 | 1.7 | | 1.3 | 2.3 | | 1.6 | 0.0 | | 0.3 | 0.0 | | 0.8 | | 0.0 | 1.1 | 0.0 | | 1.4 | 0.0 | | 1.7 |
|  | | 300 | 0.1 | | 0.2 | 0.6 | | 0.4 | | 1.1 | 0.6 | 1.7 | | 0.8 | 2.2 | | 0.9 | 0.0 | | 0.2 | 0.0 | | 0.4 | | 0.0 | 0.6 | 0.0 | | 0.8 | 0.0 | | 1.0 |
|  | | 500 | 0.1 | | 0.1 | 0.6 | | 0.3 | | 1.1 | 0.5 | 1.7 | | 0.6 | 2.3 | | 0.7 | 0.0 | | 0.1 | 0.0 | | 0.3 | | 0.0 | 0.5 | 0.0 | | 0.6 | 0.0 | | 0.7 |

# Table S3. Empirical bootstrap confidence intervals (95%) for relative bias on overall survival due to censuring or excluding lost to follow-up by cancer localization, lost to follow-up percent, registry size, and follow-up time

Censoring lost to follow-up

Cancer localization Follow time (y)

Registry size: 100 cases Registry size: 300 cases Registry size: 500 cases

Lost to follow-up Lost to follow-up Lost to follow-up

|  | | 1% | 5% | 10% | 15% | 20% |  | 1% | 5% | 10% | 15% | 20% |  | 1% | 5% | 10% | 15% | 20% |
| --- | --- | --- | --- | --- | --- | --- | --- | --- | --- | --- | --- | --- | --- | --- | --- | --- | --- | --- |
| **Stomach** | 1 | 0.0 - 3.3 | 1.2 - 14.1 | 5.2 - 25.6 | 9.9 - 36.8 | 14.2 - 47.7 |  | 0.4 - 3.2 | 4.3 - 11.5 | 9.9 - 21.5 | 16.2 - 31.5 | 21.9 - 41.2 |  | 0.5 - 2.8 | 5.1 - 10.8 | 11.6 - 20.3 | 17.8 - 29.6 | 24.5 - 39.2 |
|  | 3 | 0.6 - 8.3 | 5.5 - 33.2 | 12.1 - 62.1 | 18.0 - 91.3 | 26.4 - 121.2 |  | 1.9 - 6.6 | 12.1 - 27.3 | 25.0 - 52.2 | 39.2 - 77.3 | 52.3 - 102.3 |  | 2.3 - 6.1 | 13.8 - 25.5 | 28.9 - 49.4 | 44.0 - 73.4 | 59.3 - 97.4 |
|  | 5 | 0.2 - 12.7 | 1.4 - 50.5 | 13.4 - 95.9 | 15.8 - 140.8 | 17.8 - 185.7 |  | 3.0 - 9.9 | 16.7 - 40.9 | 36.6 - 78.6 | 54.8 - 117.9 | 72.6 - 156.0 |  | 3.5 - 8.7 | 19.9 - 38.0 | 41.3 - 75.0 | 62.3 - 110.7 | 84.0 - 147.4 |
| **Colon** | 1 | 0.0 - 1.0 | 0.0 - 4.8 | 0.0 - 8.4 | 0.9 - 11.9 | 2.3 - 15.4 |  | 0.0- 0.9 | 0.5 - 3.9 | 2.0 - 7.2 | 3.7 - 10.4 | 5.5 - 13.3 |  | 0.0 - 0.9 | 0.9 - 3.6 | 2.6 - 6.7 | 4.5 - 9.6 | 6.4 - 12.4 |
|  | 3 | 0.0 - 2.0 | 0.0 - 9.8 | 2.1 - 16.3 | 4.7 - 23.1 | 7.7 - 29.9 |  | 0.0 - 1.9 | 2.1 - 7.4 | 5.5 - 13.5 | 9.1 - 19.5 | 12.7 - 25.6 |  | 0.0 - 1.9 | 2.7 - 6.9 | 6.5 - 12.7 | 10.3 - 18.4 | 14.3 - 24.1 |
|  | 5 | 0.0 - 3.4 | 1.5 - 14.8 | 5.6 - 26.6 | 10.2 - 38.2 | 14.5 - 49.3 |  | 0.4 - 3.4 | 4.5 - 12.1 | 10.4 - 22.4 | 16.4 - 32.7 | 22.8 - 42.7 |  | 0.5 - 2.8 | 5.4 - 11.2 | 12.0 - 21.0 | 18.6 - 30.9 | 25.6 - 40.5 |
| **Thyroid gland** | 1 | 0.0 - 0.1 | 0.0 - 0.4 | 0.0 - 0.9 | 0.0 - 1.4 | 0.0 - 1.8 |  | 0.0 - 0.1 | 0.0 - 0.5 | 0.0 - 0.9 | 0.0 - 1.4 | 0.0 - 1.8 |  | 0.0- 0.1 | 0.0 - 0.5 | 0.0 - 0.9 | 0.0 - 1.4 | 0.0 - 1.6 |
|  | 3 | 0.0 - 0.1 | 0.0 - 0.7 | 0.0 - 1.4 | 0.0 - 2.1 | 0.0 - 2.8 |  | 0.0 - 0.1 | 0.0 - 0.7 | 0.0 - 1.4 | 0.0 - 2.1 | 0.0 - 2.4 |  | 0.0 - 0.1 | 0.0 - 0.7 | 0.0 - 1.4 | 0.0 - 1.9 | 0.2 - 2.3 |
|  | 5 | 0.0 - 0.2 | 0.0 - 1.1 | 0.0 - 2.2 | 0.0 - 3.4 | 0.0 - 4.5 |  | 0.0 - 0.2 | 0.0 - 1.1 | 0.0 - 2.2 | 0.0 - 3.0 | 0.3 - 3.7 |  | 0.0 - 0.2 | 0.0 - 1.1 | 0.1 - 2.0 | 0.4 - 2.7 | 0.8 - 3.5 |

Negative lower limits were truncated to zero because censuring can never lead to underestimate survival

Excluding lost to follow-up

Cancer localization Follow time (y)

Registry size: 100 cases Registry size: 300 cases Registry size: 500 cases

Lost to follow-up Lost to follow-up Lost to follow-up

|  | | 1% | 5% | 10% | 15% | 20% |  | 1% | 5% | 10% | 15% | 20% |  | 1% | 5% | 10% | 15% | 20% |
| --- | --- | --- | --- | --- | --- | --- | --- | --- | --- | --- | --- | --- | --- | --- | --- | --- | --- | --- |
| **Stomach** | 1 | -1.0 - 2.1 | -5.4 - 5.8 | -8.2 - 8.7 | -10.8 - 10.8 | -12.5 - 12.5 |  | -1.0 - 1.6 | -3.3 - 3.4 | -4.7 - 4.8 | -5.9 - 6.3 | -7.0 - 7.3 |  | -1.0 - 1.2 | -2.5 - 2.6 | -3.5 - 3.8 | -4.6 - 4.6 | -5.6 - 5.6 |
|  | 3 | -1.1 - 5.3 | -5.3 - 10.5 | -11.2 - 14.9 | -17.6 - 18.6 | -18.6 - 21.2 |  | -1.0 - 2.6 | -5.3 - 5.6 | -7.2 - 7.8 | -9.2 - 10.0 | -10.9 - 11.6 |  | -1.0 - 2.0 | -4.0 - 4.4 | -5.6 - 6.2 | -7.1 - 7.6 | -8.4 - 8.8 |
|  | 5 | -1.1 - 7.3 | -5.2 - 13.9 | -11.1 - 19.2 | -18.2 - 21.0 | -24.7 - 26.8 |  | -1.0 - 3.7 | -5.3 - 7.0 | -8.5 - 10.0 | -11.0 - 12.5 | -13.3 - 14.5 |  | -1.0 - 2.5 | -4.0 - 5.5 | -6.7 - 7.6 | -8.7 - 9.2 | -10.6 - 11.2 |
| **Colon** | 1 | -1.0 - 0.7 | -3.7 - 2.8 | -4.6 - 4.3 | -5.9 - 5.5 | -6.9 - 6.6 |  | -1.0 - 0.5 | -1.9 - 1.7 | -2.7 - 2.5 | -3.4 - 3.2 | -3.9 - 3.9 |  | -0.7 - 0.5 | -1.4 - 1.3 | -2.1 - 2.0 | -2.5 - 2.5 | -3.0 - 3.1 |
|  | 3 | -1.0 - 1.4 | -5.4 - 4.4 | -6.5 - 6.5 | -8.1 - 8.2 | -9.9 - 9.8 |  | -1.0 - 1.1 | -2.5 - 2.5 | -3.7 - 3.6 | -4.8 - 4.6 | -5.7 - 5.7 |  | -1.0 - 0.9 | -2.0 - 2.0 | -2.9 - 2.8 | -3.6 - 3.6 | -4.4 - 4.3 |
|  | 5 | -1.0 - 2.2 | -5.2 - 6.2 | -8.1 - 9.1 | -10.5 - 11.2 | -12.5 - 12.9 |  | -1.0 - 1.7 | -3.3 - 3.5 | -4.9 - 5.0 | -6.1 - 6.3 | -7.4 - 7.6 |  | -1.0 - 1.2 | -2.5 - 2.7 | -3.7 - 3.7 | -4.7 - 4.8 | -5.6 - 5.8 |
| **Thyroid gland** | 1 | -1.0 - 0.1 | -1.0 - 0.4 | -1.8 - 0.9 | -2.0 - 1.4 | -2.4 - 1.6 |  | -0.3 - 0.1 | -0.8 - 0.3 | -1.0 - 0.6 | -1.1 - 0.9 | -1.3 - 1.0 |  | -0.2 - 0.1 | -0.5 - 0.3 | -0.7 - 0.5 | -0.8 - 0.7 | -1.0 - 0.9 |
|  | 3 | -1.0 - 0.1 | -1.8 - 0.6 | -1.8 - 1.3 | -2.5 - 1.7 | -3.0 - 2.2 |  | -0.3 - 0.1 | -0.8 - 0.5 | -1.1 - 0.9 | -1.3 - 1.1 | -1.6 - 1.4 |  | -0.4 - 0.1 | -0.6 - 0.4 | -0.8 - 0.7 | -1.0 - 0.9 | -1.2 - 1.1 |
|  | 5 | -1.0 - 0.2 | -1.8 - 0.9 | -2.5 - 1.6 | -3.1 - 2.4 | -3.6 - 3.1 |  | -0.6 - 0.2 | -1.0 - 0.7 | -1.3 - 1.1 | -1.7 - 1.5 | -2.0 - 1.8 |  | -0.3 - 0.1 | -0.8 - 0.6 | -1.0 - 0.9 | -1.3 - 1.1 | -1.5 - 1.4 |

Table S4. Summary of relative bias on 1-, 3- and 5- year net survival due to censuring or excluding lost to follow-up by cancer localization, lost to follow-up percent and registry size

Positive values mean overestimation bias

**1 year Censoring lost to follow-up Excluding lost to follow-up**

| **Cancer localization** | |  | | | | |  | | | Lost to follow-up | | | | |  | | | |  | | | |  |  | | | |  | | | Lost to follow-up | | | | |  | | | | | | | |
| --- | --- | --- | --- | --- | --- | --- | --- | --- | --- | --- | --- | --- | --- | --- | --- | --- | --- | --- | --- | --- | --- | --- | --- | --- | --- | --- | --- | --- | --- | --- | --- | --- | --- | --- | --- | --- | --- | --- | --- | --- | --- | --- | --- |
|  | | 1% | | | | | 5% | | | 10% | | | | | 15% | | | | 20% | | | |  | 1% | | | | 5% | | | 10% | | | | | 15% | | | | 20% | | | |
|  | | Registry size (n | | mean | SD |  | mean | | SD |  | | mean | SD |  | mean | | SD |  | mean | | SD |  |  | | mean | SD |  | mean | | SD |  | | mean | SD |  | mean | | SD |  | mean | | SD |  |
| **Stomach** | | 100 | | 1.7 |  | 1.4 | 8.8 | |  | 3.5 | | 17.6 |  | 5.6 | 26.2 | |  | 7.5 | 35.2 | |  | 9.2 |  |  | 0.0 |  | 1.3 | 0.0 | |  | 3.1 | | 0.0 |  | 4.4 | -0.1 | |  | 5.6 | -0.1 | |  | 6.7 |
|  | | 300 | | 1.7 |  | 0.8 | 8.6 | |  | 2.0 | | 17.2 |  | 3.1 | 25.8 | |  | 4.2 | 34.4 | |  | 5.2 |  |  | 0.0 |  | 0.8 | 0.0 | |  | 1.7 | | 0.0 |  | 2.5 | 0.0 | |  | 3.2 | 0.0 | |  | 3.8 |
|  | | 500 | | 1.7 |  | 0.6 | 8.6 | |  | 1.5 | | 17.2 |  | 2.4 | 25.7 | |  | 3.2 | 34.4 | |  | 4.0 |  |  | 0.0 |  | 0.6 | 0.0 | |  | 1.3 | | 0.0 |  | 1.9 | 0.0 | |  | 2.4 | 0.0 | |  | 2.9 |
| **Colon** | | 100 | | 0.5 | 0.7 | | 2.7 | | 1.7 | | | 5.4 | 2.5 | | 8.2 | | 3.2 | | 11.1 | | 3.8 | |  | | 0.0 | 0.7 | | 0.0 | | 1.7 | | | 0.0 | 2.4 | | 0.0 | | 3.1 | | 0.1 | | 3.7 | |
|  | | 300 | | 0.5 | 0.4 | | 2.7 | | 1.0 | | | 5.4 | 1.4 | | 8.2 | | 1.8 | | 10.9 | | 2.2 | |  |  | 0.0 | 0.4 | | 0.0 | | 1.0 | | | 0.0 | 1.4 | | 0.0 | | 1.8 | | 0.0 | | 2.1 | |
|  | | 500 | | 0.5 | 0.3 | | 2.7 | | 0.8 | | | 5.4 | 1.1 | | 8.1 | | 1.4 | | 10.9 | | 1.7 | |  |  | 0.0 | 0.3 | | 0.0 | | 0.8 | | | 0.0 | 1.1 | | 0.0 | | 1.4 | | 0.0 | | 1.6 | |
| **Thyroid gland** | | 100 | | 0.0 | 0.2 | | 0.2 | | 0.5 | | | 0.5 | 0.7 | | 0.7 | | 0.9 | | 1.0 | | 1.0 | |  | | 0.0 | 0.2 | | 0.0 | | 0.5 | | | 0.0 | 0.7 | | 0.0 | | 0.9 | | 0.0 | | 1.1 | |
|  | | 300 | | 0.1 | 0.1 | | 0.2 | | 0.3 | | | 0.5 | 0.4 | | 0.7 | | 0.5 | | 1.0 | | 0.6 | |  |  | 0.0 | 0.1 | | 0.0 | | 0.3 | | | 0.0 | 0.4 | | 0.0 | | 0.5 | | 0.0 | | 0.6 | |
|  | | 500 | | 0.0 | 0.1 | | 0.2 | | 0.2 | | | 0.5 | 0.3 | | 0.7 | | 0.4 | | 1.0 | | 0.4 | |  |  | 0.0 | 0.1 | | 0.0 | | 0.2 | | | 0.0 | 0.3 | | 0.0 | | 0.4 | | 0.0 | | 0.5 | |

**3 year Censoring lost to follow-up Excluding lost to follow-up**

| **Cancer localization** | |  | | | |  | | | Lost to follow-up | | | | |  | | | |  | |  |  | | | |  | | | Lost to follow-up | | | | |  | | | | | | |
| --- | --- | --- | --- | --- | --- | --- | --- | --- | --- | --- | --- | --- | --- | --- | --- | --- | --- | --- | --- | --- | --- | --- | --- | --- | --- | --- | --- | --- | --- | --- | --- | --- | --- | --- | --- | --- | --- | --- | --- |
|  | | 1% | | | | 5% | | | 10% | | | | | 15% | | | | 20% | |  | 1% | | | | 5% | | | 10% | | | | | 15% | | | | 20% | | |
|  | | Registry size (n mean | | SD |  | mean | | SD |  | | mean | SD |  | mean | | SD |  | mean | | SD |  | mean | SD |  | mean | | SD |  | | mean | SD |  | mean | | SD |  | mean | | SD |
| **Stomach** | | 100 4.5 | |  | 2.6 | 22.6 | |  | 8.0 | | 45.0 |  | 14.3 | 67.8 | |  | 20.9 | 90.4 | | 26.7 |  | 0.0 |  | 2.1 | 0.0 | |  | 4.9 | | 0.0 |  | 7.1 | 0.1 | |  | 9.0 | -0.1 | | 10.6 |
|  | | 300 4.4 | |  | 1.4 | 21.8 | |  | 4.3 | | 43.6 |  | 7.7 | 65.5 | |  | 10.9 | 87.2 | | 14.1 |  | 0.0 |  | 1.2 | 0.0 | |  | 2.7 | | 0.0 |  | 4.0 | 0.0 | |  | 5.0 | 0.1 | | 5.9 |
|  | | 500 4.3 | |  | 1.1 | 21.6 | |  | 3.3 | | 43.2 |  | 5.8 | 64.9 | |  | 8.3 | 86.6 | | 10.8 |  | 0.0 |  | 0.9 | 0.0 | |  | 2.1 | | 0.0 |  | 3.0 | 0.0 | |  | 3.9 | 0.0 | | 4.6 |
| **Colon** | | 100 1.1 | |  | 1.1 | 5.6 | |  | 2.7 | | 11.3 |  | 4.1 | 17.0 | |  | 5.4 | 22.6 | | 6.5 |  | 0.0 |  | 1.1 | 0.0 | |  | 2.4 | | 0.0 |  | 3.5 | 0.0 | |  | 4.4 | 0.1 | | 5.2 |
|  | | 300 1.1 | |  | 0.7 | 5.5 | |  | 1.5 | | 11.1 |  | 2.3 | 16.7 | |  | 3.0 | 22.3 | | 3.6 |  | 0.0 |  | 0.6 | 0.0 | |  | 1.4 | | 0.0 |  | 2.0 | 0.0 | |  | 2.5 | 0.0 | | 3.0 |
|  | | 500 1.1 | |  | 0.5 | 5.6 | |  | 1.2 | | 11.1 |  | 1.8 | 16.6 | |  | 2.3 | 22.2 | | 2.8 |  | 0.0 |  | 0.5 | 0.0 | |  | 1.1 | | 0.0 |  | 1.6 | 0.0 | |  | 1.9 | 0.0 | | 2.3 |
| **Thyroid gland** | | 100 0.1 | |  | 0.3 | 0.4 | |  | 0.7 | | 0.7 |  | 0.9 | 1.1 | |  | 1.1 | 1.5 | | 1.3 |  | 0.0 |  | 0.3 | 0.0 | |  | 0.6 | | 0.0 |  | 0.9 | 0.0 | |  | 1.1 | 0.0 | | 1.3 |
|  | | 300 0.1 | |  | 0.2 | 0.4 | |  | 0.4 | | 0.8 |  | 0.5 | 1.1 | |  | 0.7 | 1.5 | | 0.8 |  | 0.0 |  | 0.2 | 0.0 | |  | 0.4 | | 0.0 |  | 0.5 | 0.0 | |  | 0.7 | 0.0 | | 0.8 |
|  | | 500 0.1 | |  | 0.1 | 0.4 | |  | 0.3 | | 0.8 |  | 0.4 | 1.1 | |  | 0.5 | 1.5 | | 0.6 |  | 0.0 |  | 0.1 | 0.0 | |  | 0.3 | | 0.0 |  | 0.4 | 0.0 | |  | 0.5 | 0.0 | | 0.6 |

**5 year Censoring lost to follow-up Excluding lost to follow-up**

| **Cancer localization** | |  | | | |  | | | Lost to follow-up | | | | |  | | | |  | | |  | |  | | | |  | | | Lost to follow-up | | | | |  | | | | | | |
| --- | --- | --- | --- | --- | --- | --- | --- | --- | --- | --- | --- | --- | --- | --- | --- | --- | --- | --- | --- | --- | --- | --- | --- | --- | --- | --- | --- | --- | --- | --- | --- | --- | --- | --- | --- | --- | --- | --- | --- | --- | --- |
|  | | 1% | | | | 5% | | | 10% | | | | | 15% | | | | 20% | | |  | | 1% | | | | 5% | | | 10% | | | | | 15% | | | | 20% | | |
|  | | Registry size (n mean | | SD |  | mean | | SD |  | | mean | SD |  | mean | | SD |  | mean | | SD |  | | mean | | SD |  | mean | | SD |  | | mean | SD |  | mean | | SD |  | mean | | SD |
| **Stomach** | | 100 7.1 | |  | 4.3 | 35.9 | |  | 15.0 | | 71.7 |  | 28.4 | 107.1 | |  | 40.0 | 143.3 | | 53.8 |  |  | 0.0 | |  | 2.7 | 0.1 | |  | 6.1 | | 0.0 |  | 8.8 | -0.1 | |  | 11.2 | -0.1 | | 13.4 |
|  | | 300 6.7 | |  | 2.3 | 33.6 | |  | 7.2 | | 67.3 |  | 13.5 | 100.8 | |  | 19.2 | 134.5 | | 24.7 |  |  | 0.0 | |  | 1.5 | 0.0 | |  | 3.4 | | 0.0 |  | 5.0 | 0.0 | |  | 6.2 | -0.1 | | 7.5 |
|  | | 500 6.7 | |  | 1.7 | 33.3 | |  | 5.6 | | 66.6 |  | 10.1 | 100.0 | |  | 14.8 | 132.9 | | 19.1 |  |  | 0.0 | |  | 1.1 | 0.0 | |  | 2.6 | | 0.0 |  | 3.9 | 0.0 | |  | 4.8 | -0.1 | | 5.7 |
| **Colon** | | 100 2.0 | |  | 1.7 | 10.2 | |  | 4.2 | | 20.5 |  | 6.7 | 30.5 | |  | 8.9 | 41.0 | | 11.1 |  | | 0.0 | |  | 1.4 | 0.1 | |  | 3.2 | | 0.1 |  | 4.6 | -0.1 | |  | 5.9 | 0.0 | | 6.9 |
|  | | 300 2.0 | |  | 0.9 | 10.0 | |  | 2.4 | | 20.0 |  | 3.7 | 30.0 | |  | 5.0 | 40.0 | | 6.1 |  |  | 0.0 | |  | 0.8 | 0.0 | |  | 1.8 | | 0.0 |  | 2.6 | 0.0 | |  | 3.3 | -0.1 | | 4.0 |
|  | | 500 2.0 | |  | 0.7 | 10.0 | |  | 1.8 | | 20.0 |  | 2.9 | 29.9 | |  | 3.8 | 40.0 | | 4.8 |  |  | 0.0 | |  | 0.6 | 0.0 | |  | 1.4 | | 0.0 |  | 2.0 | 0.0 | |  | 2.6 | 0.0 | | 3.1 |
| **Thyroid gland** | | 100 0.1 | |  | 0.4 | 0.7 | |  | 0.9 | | 1.3 |  | 1.3 | 2.0 | |  | 1.6 | 2.6 | | 1.9 |  | | 0.0 | |  | 0.3 | 0.0 | |  | 0.8 | | 0.0 |  | 1.2 | 0.0 | |  | 1.4 | 0.0 | | 1.7 |
|  | | 300 0.1 | |  | 0.2 | 0.6 | |  | 0.5 | | 1.3 |  | 0.7 | 1.9 | |  | 0.9 | 2.6 | | 1.1 |  |  | 0.0 | |  | 0.2 | 0.0 | |  | 0.5 | | 0.0 |  | 0.7 | 0.0 | |  | 0.8 | 0.0 | | 1.0 |
|  | | 500 0.1 | |  | 0.2 | 0.6 | |  | 0.4 | | 1.3 |  | 0.6 | 1.9 | |  | 0.7 | 2.6 | | 0.8 |  |  | 0.0 | |  | 0.2 | 0.0 | |  | 0.4 | | 0.0 |  | 0.5 | 0.0 | |  | 0.6 | 0.0 | | 0.8 |

# Table S5. Empirical bootstrap confidence intervals (95%) for relative bias on net survival due to censuring or excluding lost to follow-up by cancer localization, lost to follow-up percent, registry size, and follow-up time

**Censoring lost to follow-up**

**Cancer localization Follow time (y)**

Registry size: 100 cases Registry size: 300 cases Registry size: 500 cases

**Lost to follow-up Lost to follow-up Lost to follow-up**

|  | | 1% | 5% | 10% | 15% | 20% | 1% | 5% | 10% | 15% | 20% | 1% | 5% | 10% | 15% | 20% |
| --- | --- | --- | --- | --- | --- | --- | --- | --- | --- | --- | --- | --- | --- | --- | --- | --- |
| **Stomach** | 1 | 0.0 - 3.3 | 1.3 - 14.2 | 5.2 - 25.8 | 9.8 - 36.9 | 14.0 - 48.1 | 0.3 - 3.3 | 4.3 - 11.6 | 10.0 - 21.7 | 16.3 - 31.8 | 22.1 - 41.4 | 0.5 - 2.8 | 5.2 - 10.9 | 11.7 - 20.5 | 17.9 - 29.9 | 24.7 - 39.4 |
|  | 3 | 0.0 - 8.5 | 4.2 - 34.6 | 11.1 - 64.6 | 18.1 - 95.0 | 27.9 - 125.8 | 1.7 - 6.9 | 12.2 - 28.3 | 25.6 - 54.2 | 40.4 - 80.1 | 53.8 - 106.0 | 2.2 - 6.3 | 14.0 - 26.5 | 29.7 - 51.4 | 45.2 - 76.0 | 61.1 - 101.0 |
|  | 5 | 0.0 - 13.5 | 0.2 - 54.8 | 5.8 - 103.9 | 12.8 - 152.3 | 15.7 - 201.3 | 1.5 - 10.8 | 16.1 - 44.3 | 36.5 - 85.1 | 55.3 - 126.8 | 74.1 - 168.2 | 2.6 - 9.6 | 20.0 - 41.1 | 42.4 - 80.7 | 65.0 - 119.1 | 86.7 - 158.5 |
| **Colon** | 1 | 0.0 - 1.0 | 0.0 - 4.8 | 0.0 - 8.5 | 1.0 - 12.1 | 2.3 - 15.6 | 0.0 - 1.0 | 0.4 - 3.9 | 2.0 - 7.3 | 3.7 - 10.5 | 5.5 - 13.4 | 0.0 - 1.0 | 1.0 - 3.7 | 2.6 - 6.7 | 4.6 - 9.7 | 6.5 - 12.6 |
|  | 3 | 0.0 - 2.1 | 0.0 - 10.3 | 2.2 - 17.2 | 5.0 - 24.4 | 7.8 - 31.5 | 0.0 - 2.0 | 2.2 - 7.8 | 5.7 - 14.2 | 9.5 - 20.5 | 13.4 - 26.9 | 0.1 - 2.0 | 2.8 - 7.3 | 6.8 - 13.4 | 10.8 - 19.3 | 14.9 - 25.3 |
|  | 5 | 0.0 - 3.8 | 0.9 - 16.6 | 5.3 - 29.7 | 10.0 - 42.9 | 14.8 - 55.1 | 0.1 - 3.7 | 4.6 - 13.5 | 11.0 - 25.1 | 17.9 - 36.5 | 24.9 - 47.9 | 0.4 - 3.2 | 5.7 - 12.6 | 13.0 - 23.5 | 20.3 - 34.5 | 27.9 - 45.4 |
| **Thyroid gland** | 1 | 0.0 - 0.1 | 0.0 - 0.5 | 0.0 - 0.9 | 0.0 - 1.5 | 0.0 - 1.9 | 0.0 - 0.1 | 0.0 - 0.5 | 0.0 - 0.9 | 0.0 - 1.4 | 0.0 - 1.9 | 0.0 - 0.1 | 0.0 - 0.5 | 0.0 - 0.9 | 0.0 - 1.4 | 0.0 - 1.7 |
|  | 3 | 0.0 - 0.1 | 0.0 - 0.8 | 0.0 - 1.5 | 0.0 - 2.3 | 0.0 - 3.0 | 0.0 - 0.1 | 0.0 - 0.8 | 0.0 - 1.5 | 0.0 - 2.3 | 0.0 - 2.7 | 0.0- 0.2 | 0.0 - 0.8 | 0.0 - 1.5 | 0.0 - 2.0 | 0.3 - 2.5 |
|  | 5 | 0.0 - 0.3 | 0.0 - 1.3 | 0.0 - 2.6 | 0.0 - 4.0 | 0.0 - 5.2 | 0.0 - 0.3 | 0.0 - 1.3 | 0.0- 2.6 | 0.0 - 3.5 | 0.2 - 4.4 | 0.0 - 0.3 | 0.0 - 1.3 | 0.0 - 2.4 | 0.4 - 3.2 | 0.9 - 4.1 |

**Excluding lost to follow-up**

**Cancer localization Follow time (y)**

Registry size: 100 cases Registry size: 300 cases Registry size: 500 cases

**Lost to follow-up Lost to follow-up Lost to follow-up**

|  | | 1% | 5% | 10% | 15% | 20% | 1% | 5% | 10% | 15% | 20% | 1% | 5% | 10% | 15% | 20% |
| --- | --- | --- | --- | --- | --- | --- | --- | --- | --- | --- | --- | --- | --- | --- | --- | --- |
| **Stomach** | 1 | -1.0 - 2.2 | -5.4 - 5.8 | -8.3 - 8.7 | -10.7 - 10.7 | -12.4 - 12.6 | -1.0 - 1.6 | -3.3 - 3.4 | -4.7 - 4.8 | -5.9 - 6.3 | -7.1 - 7.3 | -1.0 - 1.2 | -2.5 - 2.6 | -3.5 - 3.8 | -4.6 - 4.6 | -5.6 - 5.7 |
|  | 3 | -1.1 - 5.2 | -5.3 - 10.6 | -11.3 - 14.8 | -17.6 - 18.5 | -18.6 - 21.4 | -1.0 - 2.7 | -5.2 - 5.7 | -7.2 - 7.8 | -9.2 - 10.0 | -11.0 - 11.8 | -1.0 - 2.0 | -3.9 - 4.4 | -5.6 - 6.2 | -7.2 - 7.7 | -8.5 - 9.0 |
|  | 5 | -1.1 - 7.7 | -5.3 - 15.0 | -11.2 - 19.7 | -18.1 - 23.4 | -24.8 - 28.1 | -1.0 - 3.8 | -5.3 - 7.4 | -8.6 - 10.5 | -11.0 - 13.0 | -13.7 - 15.2 | -1.0 - 2.7 | -4.1 - 5.6 | -6.8 - 8.0 | -8.7 - 9.6 | -11.0 - 11.6 |
| **Colon** | 1 | -1.0 - 0.7 | -3.7 - 2.8 | -4.6 - 4.4 | -5.9 - 5.4 | -6.9 - 6.5 | -1.0 - 0.6 | -1.9 - 1.7 | -2.7 - 2.5 | -3.4 - 3.3 | -3.9 - 3.9 | -0.7 - 0.5 | -1.4 - 1.3 | -2.1 - 2.0 | -2.6 - 2.5 | -3.0 - 3.0 |
|  | 3 | -1.0 - 1.5 | -5.3 - 4.4 | -6.6 - 6.6 | -8.1 - 8.2 | -10.0 - 9.8 | -1.0 - 1.1 | -2.5 - 2.5 | -3.7 - 3.6 | -4.7 - 4.7 | -5.7 - 5.7 | -1.0 - 0.9 | -2.0 - 2.0 | -2.9 - 2.9 | -3.7 - 3.6 | -4.4 - 4.3 |
|  | 5 | -1.0 - 2.5 | -5.2 - 6.5 | -8.3 - 9.3 | -10.6 - 11.6 | -12.8 - 13.3 | -1.0 - 1.6 | -3.3 - 3.6 | -4.9 - 5.1 | -6.2 - 6.4 | -7.5 - 7.9 | -1.0 - 1.2 | -2.5 - 2.7 | -3.7 - 3.9 | -4.8 - 4.9 | -5.7 - 5.8 |
| **Thyroid gland** | 1 | -1.0 - 0.1 | -1.0 - 0.5 | -1.8 - 0.9 | -2.0 - 1.4 | -2.5 - 1.7 | -0.3 - 0.1 | -0.8 - 0.3 | -1.0 - 0.6 | -1.1 - 0.9 | -1.3 - 1.1 | -0.2 - 0.1 | -0.5 - 0.3 | -0.7 - 0.5 | -0.8 - 0.7 | -1.0 - 0.9 |
|  | 3 | -1.0 - 0.2 | -1.8 - 0.7 | -1.9 - 1.3 | -2.6 - 1.8 | -3.0 - 2.3 | -0.3 - 0.1 | -0.8 - 0.5 | -1.1 - 0.9 | -1.3 - 1.2 | -1.6 - 1.4 | -0.4 - 0.1 | -0.6 - 0.4 | -0.9 - 0.7 | -1.1 - 0.9 | -1.2 - 1.1 |
|  | 5 | -1.0 - 0.4 | -1.8 - 1.1 | -2.6 - 1.8 | -3.2 - 2.5 | -3.6 - 3.1 | -0.6 - 0.2 | -1.0 - 0.7 | -1.4 - 1.2 | -1.8 - 1.5 | -2.0 - 1.9 | -0.4 - 0.2 | -0.7 - 0.6 | -1.1 - 1.0 | -1.3 - 1.2 | -1.6 - 1.4 |
